# The role of illness perceptions on medication nonadherence among patients with hypertension: A multicenter study in indonesia

**DOI:** 10.3389/fphar.2022.985293

**Published:** 2022-09-26

**Authors:** Sofa D. Alfian, Nurul Annisa, Dyah A. Perwitasari, Andre Coelho, Rizky Abdulah

**Affiliations:** ^1^ Department of Pharmacology and Clinical Pharmacy, Faculty of Pharmacy, Universitas Padjadjaran, Jatinangor, Indonesia; ^2^ Drug Utilization and Pharmacoepidemiology Research Group, Center of Excellence for Pharmaceutical Care Innovation, Universitas Padjadjaran, Jatinangor, Indonesia; ^3^ Unit Clinical Pharmacy and Community, Faculty of Pharmacy, Universitas Mulawarman, Samarinda, Indonesia; ^4^ Department of Clinical Pharmacy, Faculty of Pharmacy, Universitas Ahmad Dahlan, Yogyakarta, Indonesia; ^5^ Health and Technology Research Center (H&TRC), Escola Superior de Tecnologia da Saúde, Instituto Politécnico de Lisboa (ESTeSL), Lisbon, Portugal

**Keywords:** illness perception, medication adherence, antihypertensive medication, LMICs, Indonesia

## Abstract

**Introduction:** Nonadherence to antihypertensive medications is recognized as a significant cause of treatment failure. Therefore, identifying its underlying factors, particularly from the patient’s perspective, is essential for developing tailored intervention strategies. The objective of this study was to evaluate the associations between different domains of illness perception and medication nonadherence among patients with hypertension in Indonesia.

**Patients and methods:** A multicenter cross-sectional study was conducted among patients with hypertension aged 18 years old and older who were using antihypertensive medications in the last 3 months in the community health centers in the three cities in Indonesia. The different domains of illness perception (e.g., consequences, timeline, personal control, treatment control, identity, concerns, comprehension, and emotional response) and medication nonadherence were assessed using a validated Brief Illness Perceptions Questionnaire (BIPQ) and Medication Adherence Report Scale (MARS), respectively. A logistic regression analysis was conducted to evaluate the associations between the different domains of illness perception and medication nonadherence adjusting for confounders. The odds ratios (ORs) and 95% confidence intervals (CIs) were reported.

**Results:** A total of 440 participants were included, whom 41.8% reported nonadherence to antihypertensive medications. The majority of the participants were females (64.3%) and aged between 60 and 69 years old (39.5%). The treatment control (OR: 0.80, 95% confidence interval: 0.7–10.90), patient’s comprehension of hypertension (OR: 0.89, 95% CI: 0.820–0.97), and patient’s emotions (OR: 0.93, 95% CI: 0.88–0.99) were significantly associated with medication nonadherence. No significant associations were observed between the other domains of illness perception and medication nonadherence.

**Conclusion:** Different dimensions of illness perception were associated with non-adherence to antihypertensive medications. Educational interventions should be developed based on patients’ perception of their illness.

## 1 Introduction

Hypertension is a global public health challenge which is known as the major modifiable risk factor for the global burden of cardiovascular disease and all-cause mortality worldwide (Mills et al., 2020). The hypertension prevalence among adults was reported higher in low-income to middle-income countries (LMICs) compared to high-income countries (HICs) (Nielsen et al., 2017). Despite the increasing prevalence, however, its treatment and control remains suboptimal particularly in LMICs (Mills et al., 2020). Although the effectiveness of antihypertensive medication is well documented, less than a 10th of patients with hypertension have their blood pressure controlled (Schutte et al., 2021).

Medication nonadherence is a well-recognized determinant contributing to the uncontrolled blood pressure in patients with hypertension (Burnier and Egan, 2019). Of the 25 studies involving 12,603 patients with hypertension in the previous meta-analysis, 45.2% of the patients were reported nonadherent to antihypertensive medications (Abegaz et al., 2017). Patients may experience different types of nonadherence to antihypertensive medications, that is intentional (a conscious and active decision after balancing the pros and cons of a medication) and unintentional (passive decision due to factors beyond the patient’s control) non-adherence (Lowry et al., 2005; Bae et al., 2016). The nonadherence to antihypertensive medications is associated with increased risks of resistant hypertension (Hamrahian, 2020), cerebrovascular and coronary events (Corrao et al., 2011), reduced quality of life (Peacock et al., 2021), and increased healthcare costs (Mennini et al., 2015). Therefore, recent guidelines have highlighted the urgency to address medication nonadherence as a major problem in the management of hypertension (Unger et al., 2020).

The guidelines in Indonesia emphasized the urgency of addressing medication nonadherence during the patient counseling in the clinical practices (Indonesia, 2016a; 2016b). Yet, information of which focus needs more attention to improve medication adherence remains unclear. The previous studies have identified possible factors that are associated with nonadherence to antihypertensive medications in LMICs and HICs, such as gender (Abegaz et al., 2017), socioeconomic-related factors (van der Laan et al., 2017), therapy-related factors (Alfian et al., 2019), healthcare team and system-related factors (van der Laan et al., 2017), and patient-related factors such as medication beliefs (Alfian et al., 2020). Another important factor that contributes to nonadherence of patients with antihypertensive medications is the asymptomatic and lifelong nature of the disease (Sabate, 2003).

The common-sense model of self-regulation showed that an adaptive response to illness relies on the patient’s belief or perception (Leventhal et al., 1992). This illness perception may not be validated scientifically or medically but is developed from patient experience, social influences, and interaction with healthcare professionals (Leventhal H, Meyer D, 1980). Therefore, the patient’s perception of their illness is a decisive factor that influences their health-seeking behavior (Norfazilah et al., 2013). Patients with hypertension who were aware of their disease being chronic are more likely to take medication in order to control or prevent more severe conditions (Chen et al., 2011). The previous studies conducted in either LMICs and HICs showed that the illness perception plays a significant role on patient’s adherence to antihypertensive medications (Ross et al., 2004; Žugelj et al., 2010; Rajpura and Nayak, 2014; Taheri-Kharameh et al., 2016). However, limited study has focused on the different domains of patient’s perspective on their diseases and its impact on medication nonadherence particularly in Indonesia, where societal and racial determinants may lead to significant differences from the other countries. This information is needed to develop tailored intervention strategies. Therefore, the objective of this study was to evaluate the associations between different domains of illness perception and medication nonadherence among patients with hypertension in Indonesia.

## 2 Patients and methods

### 2.1 Study design, setting, and patient recruitment

We conducted a multicenter cross-sectional survey in Bandung City, Samarinda City, and the Special Region of Yogyakarta in Indonesia from October 2018 to September 2019. We purposively recruited patients from selected community health centers (CHCs) in accordance with the required number of patients diagnosed with hypertension. The CHCs are primary healthcare centers aimed to provide integrated chronic disease management at the subdistrict level and staffed with doctors, dentists, nurses, midwives, and pharmacists. The patients aged 18 years and older, diagnosed with hypertension for more than 1 year, taking antihypertensive medications in the last 3 months, and literate in the Indonesian language were eligible to participate in this study. We excluded patients with severe mental or physical constraints, pregnant, or in the lactation period. This study was approved by the Health Research Ethics Committee of Universitas Padjadjaran, Indonesia (No. 1137/UN6. KEP/EC/2018) and was performed according to the Declaration of Helsinki. We obtained a written informed consent from all of the patients who participated in this study.

### 2.2 Measures

#### 2.2.1 Illness perceptions

Patients’ perceptions of hypertension were measured using the Brief Illness Perceptions Questionnaire (BIPQ) (Broadbent et al., 2006). The BIPQ is a 9-item self-reported questionnaire developed to rapidly evaluate the cognitive and emotional representation of an illness (Broadbent et al., 2006). The BIPQ includes five subscales for cognitive representation of illness perception (e.g., consequences, timeline, personal control, treatment control, and identity), two subscales on emotional representation of illness perception (e.g., concern and emotions), and two subscales on illness comprehensibility of illness perception (e.g., comprehension and perceived cause of illness). However, the question on perceived cause of illness is an open-ended question that measures the patients’ beliefs about the causes of their illness and involves qualitative analysis. Thus, it was omitted in our study. The eight items are rated on a 10-point Likert scale and are evaluated using the following subscales: (1) consequences: perception of consequences of hypertension in daily life; (2) timeline: expectations about the duration of hypertension; (3) personal control: perception of the degree of personal control over hypertension; (4) treatment control: perception of the degree patients can control their hypertension in terms of received treatment; (5) identity: perceived symptoms of hypertension; (6) concern: concern about hypertension; (7) comprehension: understanding of hypertension; and (8) emotional burden: emotional burden due to hypertension. The total score of illness perception was calculated by reverse score for personal control, treatment control, and comprehension and then was added to the score of the other items. Thus, a higher BIPQ total score indicated that the patient perceives the illness as more threatening. The BIPQ demonstrated good psychometric properties, and it has been widely used among patients with different chronic conditions (Broadbent et al., 2015). The Indonesian version of BIPQ also showed to be valid and reliable (Rias et al., 2021). Consultation with experts was conducted to maintain the content validity of the Indonesian version of the BIPQ.

#### 2.2.2 Medication nonadherence

The adherence to antihypertensive medications was measured using the Medication Adherence Report Scale (MARS), which has shown good psychometric indicators and internal reliability (Chan et al., 2020). The MARS contains one item that reflects unintentional non-adherence (“I forget to take my antihypertensive medications”) and four items that largely reflect different forms of intentional non-adherence (e.g., “I alter the dose of my antihypertensive medications”) in the last 3 months on a 5-point Likert scale ranging from 1 (always), 2 (often), 3 (sometimes), 4 (rarely), to 5 (never). Nonadherence is defined *a priori* as a score of one–three on any of the items to reflect unintentional, intentional, and in part intentional non-adherence (Alfian et al., 2020). Adherence is defined as a score of four or five on all items to allow for missing or changing a dose rarely (Alfian et al., 2020). The MARS has been forward and backward translated and validated to Indonesian version and showed to be valid and reliable (Alfian and Putra, 2017).

#### 2.2.3 Sociodemographic covariates

The sociodemographic factors of patients included gender, age, education level completed (no formal education or elementary school, junior and senior high school, or university), and type of health insurance. The type of health insurance in Indonesia was classified as patients who could not afford to pay the health insurance premium (BPJS-PBI), those who could afford to pay the health insurance premium (BPJS-Non PBI), and those without any health insurance. We used a structured case report form to record the duration of hypertension (in years).

### 2.3 Data collection

We used a nonprobability purposive sampling technique to recruit patients. The pharmacists on duty screened the patient’s eligibility at the CHCs. The pharmacist then asked the researcher or trained research assistant to approach the eligible patient, to briefly describe and discuss the study to the patient, and ask the patient to provide written informed consent. Patients were asked to complete the BIPQ and MARS questionnaires independently. However, in some cases, some elderly patients who have difficulty in reading and writing the answer themselves were interviewed by the trained research assistants.

### 2.4 Sample size calculation

Nonadherence rates using the MARS questionnaire among Indonesian patients with hypertension based on a previous small-scale study ranged from 40 to 55% (Rahmadani et al., 2018; Alfian et al., 2020). Therefore, a minimum sample size of 180 patients was needed according to the formula for prediction models with a binary outcome (Peduzzi et al., 1996), which included a maximum of nine potential independent variables in the multivariate analysis and assuming a nonadherence proportion of 50%.

### 2.5 Data analysis

Descriptive statistics were performed to report the patient’s characteristics. We conducted complete-case analyses because some data were observed to be missing. A binary logistic regression was performed to evaluate the associations between the different domains of illness perception and medication nonadherence with manual backward elimination, adjusting for potential confounders in the univariate analysis. The adjusted odds ratios (ORs), 95% confidence interval (CI), *p*-values, and *R* ^2^ are reported. All statistical analyses were performed using the SPSS software version 27.0 (IBM, Armonk, NY, United States).

## 3 Results

### 3.1 Patient characteristics

A total of 440 participants (response rate of 85%) participated in this study from Bandung City (115 patients from six CHCs), Samarinda City (162 patients from five CHCs), and Special Region of Yogyakarta (163 patients from 18 CHCs). Most of the patients were females (64.3%), aged 60–69 years old (39.5%), and graduated from senior high school (46.8%) (Table 1). Around half of the patients reported nonadherence to antihypertensive medications (41.8%). Among the different domains of illness perception, the highest mean score was recorded for timeline (4.8 ± 3.2), which is followed by concerns (4.7 ± 3.5). The treatment control (2.2 ± 2.0) exhibited the lowest mean score, which is followed by the personal control (2.8 ± 2.3) (Table 1).

**TABLE 1:** Characteristics of patients (N = 440).

Characteristics	n (%)
Gender	
Male	157 (35.7)
Female	283 (64.3)
Age in years	
≤49	49 (11.1)
50–59	140 (31.8)
60–69	174 (39.5)
≥70	74 (16.8)
Missing	3 (0.7)
Health insurance type	
BPJS-PBI	242 (55.0)
BPJS-Non PBI	184 (41.8)
Without insurance	14 (3.2)
Last education level	
No formal education or elementary school	91 (20.7)
Junior high school	74 (16.8)
Senior high school	206 (46.8)
University	67 (15.2)
Missing	2 (0.5)
Time from diagnosis in years, mean (SD)	
Hypertension, mean (SD)	5.0 (4.2)
Missing	16
Illness perception, mean (SD)	
Consequences	4.4 (3.2)
Missing	1
Timeline	4.8 (3.2)
Missing	1
Personal control	2.8 (2.3)
Missing	1
Treatment control	2.2 (2.0)
Missing	1
Identity	4.3 (2.9)
Missing	1
Concerns	4.7 (3.5)
Missing	1
Comprehension	3.8 (2.7)
Missing	1
Emotional	4.3 (3.5)
Missing	2
MARS score	
Adherent	256 (58.2)
Nonadherent	184 (41.8)

Abbreviations: BPJS-PBI: patients who could not afford to pay the health insurance premium, BPJS-Non PBI: patients who could afford to pay the health insurance premium, MARS: medication adherence report scale, SD: standard deviation.

### 3.2 Associations between different domains of illness perception and medication nonadherence

Adjusted analyses were conducted for age, gender, and hypertension duration (*p*-value < 0.05). Lower treatment control (OR: 0.80, 95% CI: 0.71–0.90), lower patient’s comprehension of hypertension (OR: 0.89, 95% CI: 0.82–0.97), and higher patient’s emotions (OR: 0.93, 95% CI: 0.88–0.99) were associated significantly with the medication nonadherence (Table 2). No significant associations were observed between the other domains of illness perception and medication nonadherence.

**TABLE 2:** **Associations between different domai**ns **of illness perception and medication nonadherence**.

Illness perception	Nonadherence		
	OR*	95% CI	*p*-value
Treatment control	0.80	0.71–0.90	<0.001
Comprehension of hypertension	0.89	0.82–0.97	0.005
Emotions	0.93	0.88–0.99	0.026

Note: *Odds ratios were adjusted for gender, age, and hypertension duration. Goodness-of-fit *p*-value of the model, 0.307; R-squared, 17.5%.

AbbreviationsCI: confidence interval; OR: odds ratio.

## 4 Discussion

Among the different domains of illness perception, worry about hypertension timeline followed by patient’s concerns about hypertension was the dominant domain of illness perception reported among patients with hypertension in Indonesia. Furthermore, patients showed low treatment control and personal control over hypertension. Treatment control, patient’s comprehension of hypertension, and patient’s emotions were significantly associated with medication nonadherence No significant associations were observed between the other domains of illness perception and medication nonadherence.

In this study, we found that worry about hypertension timeline and patient’s concerns about hypertension were the dominant domain of illness perception. This is in line with a previous study conducted in India which found more emotional problems among patients with hypertension (Nivedita, 2015). This can partly be explained by the patients feeling overwhelmed with the lifelong nature of hypertension or concerned about the complexity of their treatment, which included changes in their treatment regimen, such as switching or adding medications. We further observed that patients showed low treatment control and personal control over hypertension. These patients need to be targeted for tailored intervention because they were recruited from primary healthcare settings, who are without complications and severe conditions, and relatively healthy.

We observed that the treatment control was significantly associated with medication nonadherence. A previous study conducted in Nepal showed that although patients perceived hypertension as highly threatening, those who have a strong belief that medication will cure their hypertension and prevent complications were reported more adherent to medications (Shakya et al., 2020). Although these patients perceive hypertension as a chronic disease, they believe that it can be controlled with regular medication and behavior modification (Ross et al., 2004; Maharjan et al., 2017). Thus, the perception of treatment effectiveness is important because it can predict adherence behavior (Žugelj et al., 2010). This is further supported by a mixed-method study conducted in Malaysia, Hong Kong, South Korea, Taiwan, Indonesia, Thailand, and Philippines, which revealed that patients strongly believed hypertension can be controlled by taking medications but they were resistant to lifestyle modification (Rahman et al., 2015).

We further observed that patient’s comprehension of hypertension was significantly associated with medication nonadherence. This is in accordance with the previous studies conducted in Iran (Taheri-Kharameh et al., 2016) and Nepal (Shakya et al., 2020). The previous studies conducted in Hongkong and India showed that poor understanding of illness is common among patients with hypertension (Nivedita, 2015; Lo et al., 2016). Our finding may be related to the asymptomatic nature of hypertension. Furthermore, patients might forget any information obtained from the healthcare professional or that the information available was not tailored for managing their hypertension (Lo et al., 2016). Although some Asian patients reported good understanding of the causes and consequences of hypertension, yet, lack of urgency to control their blood pressure was also reported (Rahman et al., 2015). Therefore, a comprehensive information regarding hypertension from healthcare professionals is needed to address this gap.

While treatment control refers to a cognitive aspect, however, concern and emotional burden due to hypertension refer to emotional aspects of illness representations (Broadbent et al., 2006). We observed that patients’ emotions were also significantly associated with medication nonadherence. This is in line with the previous studies conducted in either LMICs and HICs which reported a strong emotional perception affecting nonadherence to antihypertensive medications (Ross et al., 2004; Žugelj et al., 2010; Hsiao et al., 2012). An emotional response may negatively influence medication adherence by stimulating maladaptive coping mechanisms, for example, denial (Ross et al., 2004). Therefore, those who can control their stress levels effectively will show fewer concerns regarding illness and treatment which may lead to better medication adherence. No significant associations were observed between the other domains of illness perception and medication nonadherence.

The strength of this study is that we analyzed the similarities and differences in associations of different domains of illness perception with medication nonadherence were. Therefore, we were able to provide information on which specific domain of illness perception was associated with medication nonadherence and requires further attention from the healthcare professionals. Furthermore, the high response rate in this study illustrates that our findings are generalizable for patients with hypertension in Indonesia who visit the CHCs. The generalizability of our findings was further strengthened by the fact that this study was conducted as a multicenter survey in the three different main cities in Indonesia.

However, some limitations need to be addressed. A nonprobability purposive sampling technique was used to collect data. As a consequence, our findings may be prone to some volunteer bias. We also may have underestimated patients’ perceptions about hypertension and overestimated their medication adherence since most of them who participated in this study were those who visited the CHCs regularly and without complications. Therefore, our findings may represent the illness perceptions and medication adherence in relatively healthier patients with hypertension. The overestimated medication adherence in our study may also be due to social desirability, recall bias, and the use of subjective assessment of medication adherence. However, the data were obtained by trained research assistants using a predefined standard protocol to obtain more reliable data. Furthermore, no causal association between illness perceptions and medication nonadherence can be made due to the cross-sectional study design. We also could not capture the dynamic nature of illness perception and medication adherence as these constructs may change over time. Thus, we could not measure any changes in the trend of these constructs over time. Furthermore, previous systematic reviews showed that the evidences regarding the association between illness perceptions and medication adherence in patients with chronic diseases are inconclusive (Kucukarslan, 2012; Shahin et al., 2019; Oliveira et al., 2022). Therefore, our findings may also be confounded by unmeasured factors such as comorbidity, number of medications, distress, treatment satisfaction (Saarti et al., 2015), and medication belief (Alfian et al., 2020). Further studies are needed to evaluate the association between different domains of illness perception and medication nonadherence in uncontrolled patients with hypertension employing longitudinal design and a more objective assessment of adherence, such as medication event monitoring system (MEMS). In addition, further studies controlling for other factors not covered in this study are important to develop effective tailored interventions in the clinical practice.

Our findings emphasized the urgent need for developing interventions to modify patients’ perceptions not only about hypertension but also about their treatment in order to improve their medication adherence. These could be tailored interventions taking into consideration that illness perceptions can be changed by educational interventions (Petrie and Weinman, 2006). Furthermore, our findings showed that although patients perceived that hypertension is a chronic disease which may lead to some severe complications and emotional burdens, patients also perceived that hypertension could be controlled by medication and demonstrated an understanding of the illness.

## 5 Conclusion

Different dimensions of illness perception were associated with non-adherence to antihypertensive medications. The healthcare providers need to pay more attention to patients’ illness perceptions, including their treatment control, comprehension of hypertension, and negative emotional response. Therefore, educational interventions should be developed based on patients’ perception of their illness.

## Data Availability

The raw data supporting the conclusion of this article will be made available by the authors, without undue reservation.
